# Hemolytic Anemia as a Provoking Factor for Recurrent Venous Thromboembolism: A Case Report

**DOI:** 10.7759/cureus.54361

**Published:** 2024-02-17

**Authors:** Pablo Demelo-Rodríguez, Sabela Castañeda-Pérez, Leyre Alonso-Gonzalo, Sergio Moragón-Ledesma, Francisco Galeano-Valle

**Affiliations:** 1 Venous Thromboembolism Unit, Internal Medicine, Gregorio Marañón University Hospital, Madrid, ESP; 2 Lipids and Vascular Risk, Sanitary Research Institute Gregorio Marañón, Madrid, ESP; 3 School of Medicine, Universidad Complutense de Madrid, Madrid, ESP

**Keywords:** deep vein thrombosis, acenocoumarol, anticoagulation, venous thromboembolism, warm antibody autoimmune hemolytic anemia

## Abstract

Warm antibody autoimmune hemolytic anemia (WAIHA) is a rare disease that leads to the destruction of red blood cells in the reticuloendothelial system through the mediation of agglutinins (immunoglobulin G (IgG) type in most cases) that attach to the erythrocyte wall at 37 °C. The association of WAIHA and venous thromboembolism (VTE) seems to be higher than other hemolytic disorders classically associated with VTE and there is a current investigation aimed at clarifying this association and establishing some criteria to use anticoagulant treatment in patients with WAIHA. Despite this, WAIHA is a rare cause for the development of recurrent VTE under secondary prophylactic anticoagulant treatment, with only a few cases described in the literature. We present the case of a patient who developed a recurrence of deep vein thrombosis during a WAIHA episode despite treatment with acenocoumarol and a review of the literature.

## Introduction

Warm antibody autoimmune hemolytic anemia (WAIHA) is a disease of low prevalence (0.1-0.3%) with a predominance in females. It leads to the destruction of red blood cells in the reticuloendothelial system through the mediation of agglutinins (immunoglobulin G (IgG) type in most cases) that attach to the erythrocyte wall at 37 °C [1]. Clinically, WAIHA presents as asthenia, fever, weight loss, arthralgia, abdominal pain, chest pain, and dyspnea [2]. Most cases of WAIHA are idiopathic, although an association has been found with systemic diseases such as systemic lupus erythematosus [3] and lymphoproliferative syndromes, especially chronic lymphocytic leukemia [4,5]. The treatment of choice is corticosteroids, and the response can be delayed several weeks, with a relapse rate of 25-50% per year [1].

Venous thromboembolism (VTE) comprises pulmonary embolism (PE) and deep vein thrombosis (DVT). The most frequent provoking factors are prolonged immobilization, surgery, and neoplasms, although 50% of patients have no provoking factor [6]. Anticoagulant treatment is highly effective in preventing recurrences [7]. The most frequent causes of VTE recurrence are poor international normalized ratio (INR) control, active cancer, and inflammatory diseases. WAIHA is a rare cause of VTE recurrence.

We present the case of an 83-year-old male patient who developed a recurrence of DVT during a WAIHA episode despite secondary prophylactic treatment with acenocoumarol, and we review the scientific evidence. This case aims to present a rare case of recurrent VTE during a WAIHA episode. Investigating the reasons for this association could help prevent VTE recurrences during a WAIHA crisis in these patients.

## Case presentation

**Figure 1:**
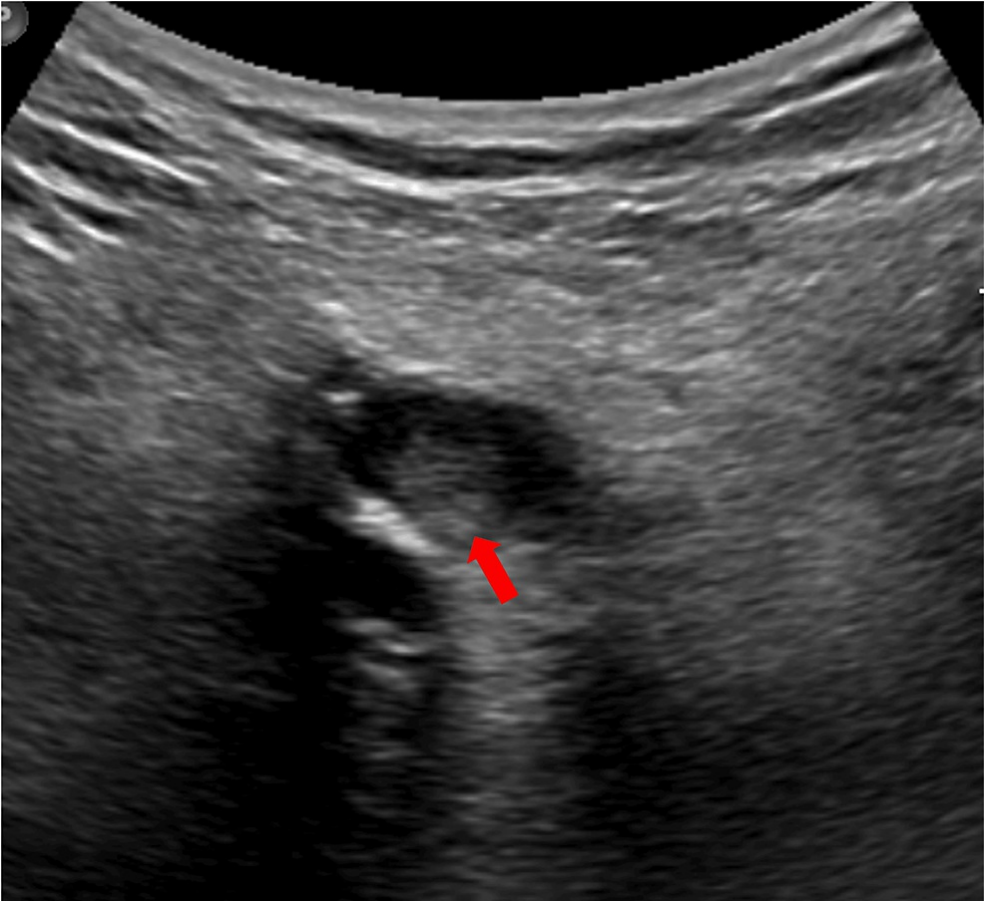
Ultrasound of the left lower limb Compression ultrasound of the left lower limb showing echogenic material (red arrow) and an absence of collapse upon compression in the popliteal vein

**Figure 2:**
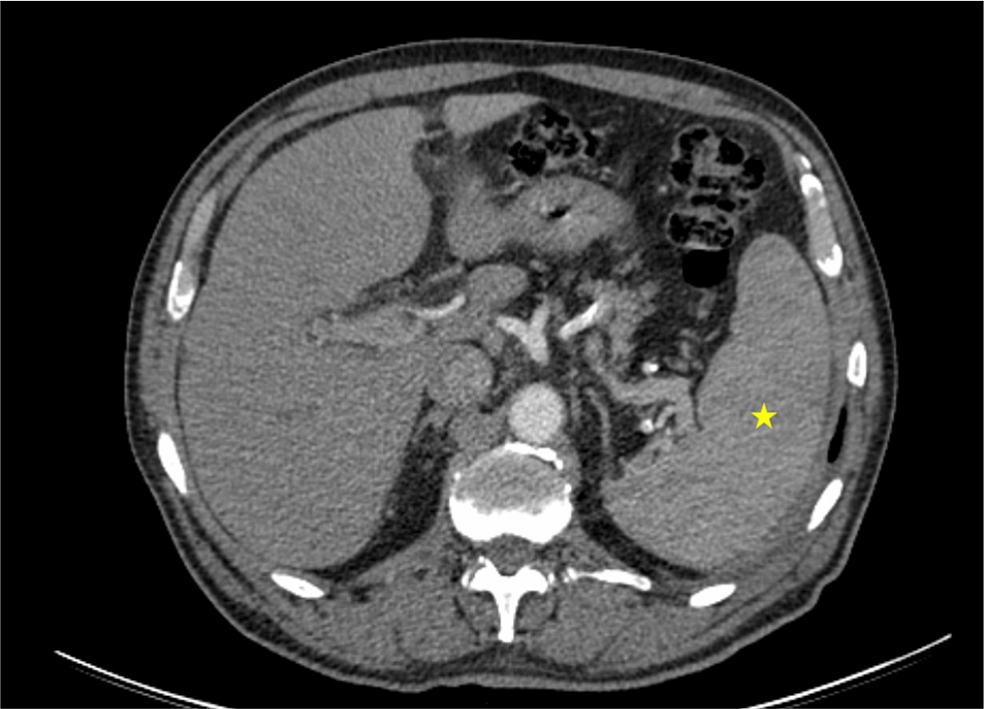
Abdominal CT Abdominal CT scan showing a 15 cm splenomegaly (yellow star), with no other significant findings

We present the case of an 83-year-old male with a history of arterial hypertension and an episode of unprovoked DVT in the right lower limb two years before, for which he was under treatment with acenocoumarol with adequate INR control (time in therapeutic range 90%). He went to the emergency department for edema and pain in the left lower limb (LLL) along with intense fatigue and dyspnea on exertion. He had no history of long travel, immobilization, or surgery in the previous three months. Upon arrival, blood pressure was 124/75 mmHg, heart rate 75 bpm, and left lower limb showed edema. The remainder of the physical examination was normal. The results of the laboratory tests are shown in Table 1. A peripheral blood smear revealed anisocytosis with macroovalocytes and neutrophil hyposegmentation. An ultrasound confirmed the diagnosis of DVT in LLL affecting the popliteal vein (Figure 1). A thorax-abdomen-pelvis computed tomography (CT) showed a splenomegaly of 15 cm (Figure 2). Gastroscopy, colonoscopy, bone marrow biopsy, and tumor markers were normal. Hepatitis B and C virus serologies were negative. The autoimmunity study revealed positive antinuclear antibodies (1/320), polyclonal hypergammaglobulinemia (IgG 2,510 mg/dL, IgM 690 mg/dl), and direct positive Coombs test for IgG, C3 76 mg/dL, and C4 15.9 mg/dL, establishing the diagnosis of WAIHA. Treatment with oral prednisone 80 mg daily was initiated (patient's weight was 88 kg) with significant improvement in symptoms and laboratory abnormalities. The anticoagulant treatment was transiently switched to tinzaparin 14,000 IU/day for 12 days and, subsequently, acenocoumarol was reintroduced. Four years after the episode, the patient has remained without new WAIHA episodes or VTE recurrences.

**Table 1:** Laboratory results INR: international normalized ratio. ALT: alanine transaminase. AST: aspartate aminotransferase. GGT: gamma-glutamyl transferase. LDH: lactate dehydrogenase.

Parameter	Result	Reference value	Units
Hemoglobin	10.5	13.8-17.2	g/dL
Mean corpuscular volume	103	83-97	fL
Reticulocytes	10	0-5-2.5	%
INR	1.38	0.8-1.1	-
ALT	240	7-56	U/L
AST	269	5-40	U/L
GGT	131	5-40	U/L
Alkaline phosphatase	141	44-147	U/L
Bilirubin (total)	3.6	0.1-1.2	mg/dL
Bilirubin (direct)	1.8	0.1-0.3	mg/dL
Bilirubin (indirect)	1.8	0.2-0.8	mg/dL
LDH	572	140-280	U/L
Ferritin	170	24-336	ng/mL
Transferrin	280	204-360	mg/dL
Transferrin saturation	21	20-45	%
Iron	76	60-170	mcg/dL
Haptoglobin	<6	41-165	mg/dL

## Discussion

The association of WAIHA and VTE was first proposed in 1960 [8], and since then, the prevalence of VTE in patients with WAIHA has been estimated in 15-33% in the case series published. This prevalence rate is higher than other hemolytic disorders classically associated with VTE such as paroxysmal nocturnal hemoglobinuria [9]. The state of hypercoagulability in WAIHA is probably multifactorial, including the erythrocyte fragmentation that leads to the formation of thrombin, an increase in tissue factor by proinflammatory cytokines, intravascular heme factor, and soluble CD40 ligand, as well as platelet activation due to the presence of free hemoglobin and the consumption of nitric oxide that leads to a state of vasoconstriction [10]. However, the role of WAIHA in the development of VTE is not clearly elucidated. Classically, the possible influence of the positivity of antiphospholipid antibodies or splenectomy as risk factors for thrombosis was postulated, although several recent studies have not found causation [11]. Besides, the Padua score has not demonstrated validity for the prediction of VTE risk in WAIHA. A study compared 11 patients with WAIHA and VTE vs 37 patients with WAIHA without VTE; on multivariate analysis, total bilirubin ≥ 40 μmol/L and leucocyte count above 7x109/L were significantly associated with a higher risk of thrombosis [12]. In another study including 308 patients with primary WAIHA, thrombotic events were associated with Hb levels ≤6 g/dL at onset, intravascular hemolysis, and previous splenectomy [13]. Thus, hemolytic crises are associated with the appearance of thrombotic events [10].

Although prophylaxis against VTE in patients with WAIHA is not routinely recommended, several authors establish this recommendation, especially in situations of greater risk, such as a hemolytic crisis [10], since up to 90% of the cases of VTE associated with hemolytic anemia occur in this period. Regarding PE, a delay in its diagnosis has been described due to confusion of the symptoms (severe dyspnea) with that of hemolytic anemia, however, there is no clear evidence to demonstrate the effectiveness of VTE screening during the hemolytic crisis [14].

Even if the association of WAIHA with VTE is described in the literature, the cases described in which thrombosis occurs despite anticoagulant therapy are rare. Hendrick et al. analyzed the efficacy of antithrombotic prophylaxis in patients with recurrent WAIHA and observed five cases of VTE in 15 crises, and only in 1 case of 21 of the group of patients with prophylaxis, with a chi-square with a Yacht correction of 3.29 (p <0.10) [15]. Another series reported two cases similar to ours, with one patient receiving secondary prophylaxis using low molecular weight heparin (LMWH) and the other receiving underdosed acenocoumarol (Table 2) [12]. In our case, treatment with acenocoumarol was resumed due to the lack of experience with direct oral anticoagulants in this setting and the economic limitations of the patient.

**Table 2:** Characteristics of the VTE recurrence of patients with WAIHA (case reports/case series published in the literature) *After the diagnosis of WAIHA. **The patient died five days after the episode of PE. WAIHA: warm antibody autoimmune hemolytic anemia. VTE: venous thromboembolism. DVT: deep vein thrombosis. PE: pulmonary embolism. NHL: non-Hodgkin lymphoma. LMWH: low molecular weight heparin. VKA: vitamin K antagonist. INR: international normalized ratio.

Author	Year	Age	Gender	WAIHA type	Hemolysis at VTE	DVT/PE	Padua score at diagnosis of DVT	Antithrombotic prophylaxis	Previous episode of VTE	Recurrence of VTE*
Hendrick AM (15)	2003	66	No data	Secondary (NHL)	+	PE	No data	LWMH	No data	0 **
Audia S et al. (12)	2018	42	Female	Primary	+	DVT	0	Prophylactic LWMH	No data	2
Audia S et al. (12)	2018	76	Male	Secondary (low-grade NHL)	+	DVT	2	VKA (INR 1,5)	No data	0
Our case	2019	83	Male	Primary	+	DVT	4	VKA	Yes	0

## Conclusions

In conclusion, current evidence postulates WAIHA as a risk factor for the development of VTE, regardless of the presence of antiphospholipid antibodies or splenomegaly, and places it in the spotlight as a potential criterion for the initiation of primary and/or secondary prophylaxis for VTE in the absence of other risk factors for thrombosis. More studies are needed to decipher the pathophysiology of this association and establish standardized performance criteria.
